# Enhancing Heart Failure Detection in the General Population Using the Steatosis‐Associated Fibrosis Estimator

**DOI:** 10.1002/clc.70318

**Published:** 2026-04-20

**Authors:** Wenrui Shi, Yangbin Shi, Jieun Park, Yuancong Wu

**Affiliations:** ^1^ Department of Cardiology, Fuwai Shenzhen Hospital Chinese Academy of Medical Sciences, Shenzhen, Guangdong Province China; ^2^ Department of Cardiology Shanghai Chest Hospital, Affiliated to Shanghai Jiao Tong University Shanghai China; ^3^ School of Medicine Shanghai Jiao Tong University Shanghai China; ^4^ Department of Cardiology Affiliated Hospital of Guangdong Medical University, Zhanjiang, Guangdong Province China

**Keywords:** general population, heart failure, NHANES, non‐alcoholic fatty liver disease, steatosis‐associated fibrosis estimator

## Abstract

**Purpose:**

Non‐alcoholic fatty liver disease and its fibrotic sequelae have been implicated in the development of cardiovascular complications. This study investigated the association between the steatosis‐associated fibrosis estimator (SAFE) and prevalent heart failure (HF), as well as evaluated whether SAFE could improve HF detection in the general population.

**Methods:**

A total of 33,566 participants from the 2003–2018 National Health and Nutrition Examination Survey were included. HF was identified based on self‐reported medical history.

**Results:**

The estimated HF prevalence was 3.00%. After adjusting for demographic, anthropometric, laboratory, and medical history factors, each one‐standard‐deviation increase in SAFE correlated with a 58.0% rise in HF risk. Individuals in the highest quartile of SAFE had a 2.777‐fold risk of HF compared to those in the lowest quartile. Smooth‐curve modeling revealed an almost linear association between SAFE and HF, and subgroup analyses supported this relationship across diverse populations. Incorporating SAFE into standard cardiovascular risk factors slightly improved HF discrimination (AUC: 0.873 vs. 0.876, *p* < 0.001). Reclassification metrics provided additional evidence for SAFE's incremental value.

**Conclusion:**

This study identified a nearly linear and robust relationship between SAFE and prevalent HF, suggesting that SAFE may serve as a useful indicator for enhancing HF detection in the general population.

## Graphical abstract

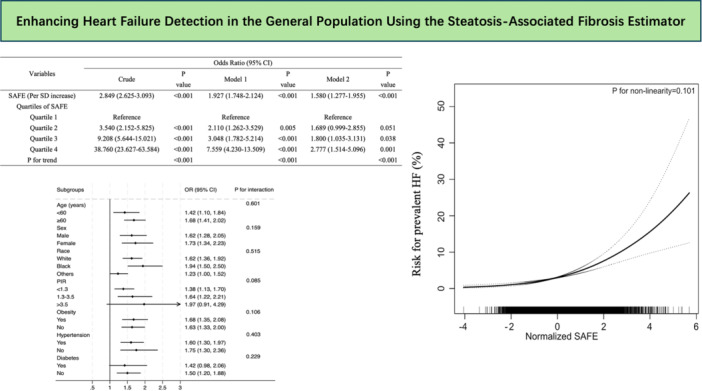

## 1 Introduction

Heart failure (HF) stands as the ultimate consequence of numerous cardiovascular conditions [1]. Over recent decades, the prevalence of HF has been steadily rising, with global estimates reaching approximately 64 million individuals affected worldwide [2]. In the United States, the prevalence of HF is projected to increase by 46% from 2012 to 2030, affecting more than 8 million adults [3]. This upward trend is not limited to developed nations; many low‐ and middle‐income countries are experiencing a significant surge in HF cases due to factors such as population aging, urbanization, and lifestyle changes [4]. The increasing prevalence of HF places a substantial strain on healthcare systems globally, highlighting the critical need for enhanced early diagnostic measures [5]. Early identification of HF within the general population, particularly in primary care settings, is essential for improving patient outcomes, reducing hospitalization rates, and alleviating the overall burden on healthcare infrastructure.

Emerging evidence highlights a significant association between Non‐alcoholic fatty liver disease (NAFLD), liver fibrosis, and HF, underscoring the interplay between hepatic and cardiac pathophysiology [6, 7]. A large cohort study found that individuals with NAFLD had an independently increased risk of developing HF, especially those with hepatic cirrhosis [8]. This relationship was also supported by a transient elastography‐based analysis, indicating that liver fibrosis severity correlates with the incidence and hospitalization rates of HF [9]. Furthermore, fibrotic severity defined by other non‐invasive methods was also associated with HF in the general population [10, 11]. Experimental research provided mechanistic insights into this association, revealing that liver fibrosis contributes to increased systemic inflammation, oxidative stress, impaired degradation of toxins, drugs, and vasoactive substances, as well as alterations in myocardial metabolism, which collectively impair cardiac function [12, 13]. Together, these epidemiological and experimental findings underscored the importance of a multidisciplinary approach in managing patients with liver fibrosis to prevent and treat concurrent HF effectively.

Traditionally, liver biopsy has been the gold standard for evaluating the extent of liver fibrosis. However, its invasive nature limits its widespread use. Recently, the Steatosis‐Associated Fibrosis Estimator (SAFE) score was developed for primary care settings to estimate clinically significant liver fibrosis in patients with NAFLD [14]. SAFE incorporates readily available variables, including age, body mass index (BMI), diabetes status, aminotransferase levels, globulin, and platelet count. A pilot study has shown that SAFE outperforms the FIB‐4 and NAFLD Fibrosis Score (NFS) in identifying low‐risk NAFLD patients and predicting survival outcomes [14]. Despite these advancements, the relationship between the SAFE score and the prevalence of heart failure (HF) remains unclear. Therefore, the current analysis aims to investigate the association between the SAFE score and prevalent HF, and to evaluate the effectiveness of SAFE in detecting prevalent HF within the general population.

## 2 Methods

### 2.1 Study Design and Population

The National Health and Nutrition Examination Survey (NHANES) is a biannual program administered by the Centers for Disease Control and Prevention (CDC) to evaluate the nation's health and dietary status. This initiative gathers data through a multistage probability sampling design, incorporating interviews, physical examinations, surveys, and laboratory tests. Before each interview and examination, participants provide informed consent. All procedures adhere to standards set by the NCHS Research Ethics Review Board, in alignment with the U.S. Department of Health and Human Services (HHS) Policy for the Protection of Human Research Subjects. Detailed explanations of NHANES methodologies and data resources are available on the CDC website (http://www.cdc.gov/nchs/nhanes/index.html).

**Figure 1:**
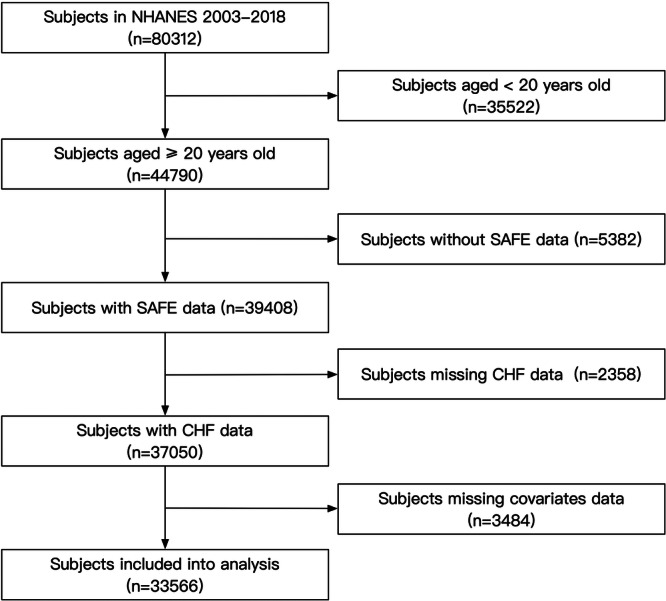
Flow chart of the subject's enrollment.

For this investigation, we drew on NHANES data from survey cycles conducted between 2003 and 2018 (totaling 80,312 participants). Individuals under the age of 20 and those lacking information on SAFE, HF, or other required covariates were omitted. After implementing these exclusions, the final sample consisted of 33,566 participants (Figure 1). All datasets analyzed in this study are freely available on the official NHANES platform.

### 2.2 Data Measurement

Data collection involved conducting interviews at participants’ residences, while laboratory evaluations were performed at the Mobile Examination Center (MEC). Trained staff gathered demographic information using a computer‐assisted interview system. Following standardized protocols, anthropometric measurements were taken, including waist circumference (WC) and height measured to the nearest 0.1 cm, and weight recorded to the nearest 0.1 kg. Blood pressure was assessed after participants had been seated and resting quietly for at least 5 min, with the average of three measurements used for analysis. Detailed procedures for blood pressure assessment are available in the “Physician Examination Procedures Manual” on the NHANES official website. All laboratory tests were conducted in CDC‐certified facilities. Fasting plasma glucose (FPG) was measured using the oxygen rate method on the Beckman DxC800 Modular Chemistry analyzer. Blood lipid levels were determined through enzymatic assays on the Roche Modular P and Roche Cobas 60,000 chemistry analyzers. Serum creatinine (Scr) was quantified using the Jaffe rate method on the DxC800 Modular Chemistry platform.

### 2.3 Outcome Ascertainment

HF was defined as answering “Yes” to the question: “Ever told you have congestive heart failure” (MCQ160E).

### 2.4 Exposure Definition

SAFE was calculated according to the following formula: SAFE = 2.97*age + 5.99*BMI (BMI > 40 was set to 40) + 62.85*diabetes (0 if absent, 1 if present) + 154.85*Ln(AST, U/L) − 58.23*Ln (ALT, U/L) + 195.48*Ln (globulin, g/dL) − 141.61*Ln (platelets, 109) – 75. BMI indicated body mass index, ALT referred to alanine aminotransferase, and AST referred to aspartate aminotransferase.

### 2.5 Covariates

Answering “Yes” to the question “Now taking prescribed medicine for hypertension” was determined as anti‐hypertensive therapy; A mean systolic blood pressure (SBP) ≥ 140 mmHg, and/or a mean diastolic blood pressure (DBP) ≥ 90 mmHg, and/or anti‐hypertensive therapy were indicated as hypertension [15]. Answering “Yes” to the question “Take diabetic pills to lower blood sugar” was determined as anti‐diabetic therapy; Based on established guidelines, diabetes was defined as FPG ≥ 7 mmol/L and/or previously diagnosed diabetes and/or self‐reported use of anti‐diabetic medication [16]. Estimated glomerular filtration rate (eGFR) was calculated according to the Chronic Kidney Disease Epidemiology Collaboration (CKD‐EPI) equation [17].

To address the intricate design of the NHANES survey, this study applied statistical weighting techniques. Categorical data were reported as frequencies accompanied by 95% confidence intervals (CIs), whereas continuous data were shown as means with their respective 95% CIs. Comparisons between groups utilized Chi‐square tests for categorical variables and *t*‐tests for continuous variables. The analysis comprised two primary sections.

Initially, the relationship between SAFE and the prevalence of HF was examined using multivariate logistic regression, presenting the findings as odds ratios (ORs) with 95% CIs. SAFE was treated both as a continuous variable, with effects measured per standard deviation (SD) increase, and as a categorical variable split into quartiles. A trend analysis (P‐for‐trend) was conducted to determine if ORs significantly decreased from the first to the fourth quartile. Covariates in the Logistic regression models were selected based on three criteria: (1) variables showed potential statistical association with frailty in univariate regression (*p* < 0.10); (2) variables that have been identified as HF‐related factors by prior literature; and (3) variables recognized as clinically relevant to HF. Additionally, a generalized additive model with spline smoothing and a logarithmic likelihood ratio test was utilized to assess the linearity of the SAFE‐HF association across its entire range. To ensure the consistency of the logistic regression outcomes, subgroup analyses were performed across various standard subpopulations.

Secondly, the study evaluated the effectiveness of SAFE in enhancing HF detection through receiver operating characteristic (ROC) analysis and reclassification metrics, including the continuous net reclassification index (NRI) and the integrated discrimination index (IDI). All statistical procedures were carried out using Stata (version 15.0), R (The R Foundation), and EmpowerStats (X&Y Solutions, Inc., Boston, MA, USA). A two‐tailed *p*‐value of less than 0.05 was considered indicative of statistical significance.

## 3 Results

Characteristic data were summarized in Table 1. Of the 33,566 participants, 1006 were diagnosed with HF. The overall mean age was 46.90 years, with HF subjects significantly older than non‐HF subjects (65.74 vs. 46.48 years, *p* < 0.001). The proportion of males was 48.73%, with a higher prevalence in the HF group (56.00% vs. 48.57%, *p* < 0.001). Racial distribution varied significantly (*p* < 0.001), with a higher proportion of non‐Hispanic Whites and Blacks in the HF group. PIR was lower in HF participants (2.31 vs. 3.05, *p* < 0.001). Table S1 showed that the differences in age, sex, and race distribution were minimal between all subjects aged ≥ 20 years and the final analytic sample. For anthropometric parameters, BMI, WC, and SBP were higher in the HF group (all *p* < 0.001). Laboratory results revealed elevated levels of FPG, triglycerides, globulin, and AST in HF subjects, while platelet, TC, LDL‐c, HDL‐c, and eGFR were lower (all *p* < 0.001). Regarding medical history, HF participants had higher rates of hypertension, diabetes, and were more likely to receive anti‐hypertensive and anti‐diabetic therapy (all *p* < 0.001). The SAFE score indicated a worse hepatic fibrosis condition in the HF group (99.69 vs. −26.34, *p* < 0.001). These findings underscore significant disparities between HF and non‐HF individuals across demographic, socioeconomic, and clinical factors.

**Table 1:** Characteristic profile of the enrolled subjects.

Variables	Total (33,566)	HF (1006)	Non‐HF (32,560)	*p* value*
Age (years)	46.90 (46.47–47.32)	65.74 (65.55–65.93)	46.48 (46.06–46.91)	< 0.001
Male (%)	48.73 (48.02–49.26)	56.00 (52.31–59.89)	48.57 (48.03–49.11)	< 0.001
Race (%)				< 0.001
Mexican American	8.22 (7.05–9.56)	3.68 (2.59–5.22)	8.32 (7.14–9.67)	
Other hispanic	5.04 (4.32–5.86)	3.75 (2.50–5.57)	5.07 (4.36–5.88)	
Non‐Hispanic white	69.29 (66.92–71.57)	74.53 (71.13–77.66)	69.18 (66.80–71.46)	
Non‐Hispanic black	10.32 (9.18–11.59)	12.77 (10.57–15.34)	10.27 (9.13–11.53)	
Others	7.13 (6.49–7.83)	5.27 (3.51–7.86)	7.17 (6.52–7.88)	
Education level (%)				< 0.001
Less than high school	15.38 (14.41–16.40)	28.10 (24.61–31.87)	15.10 (14.15–16.11)	
High school graduate/general educational development or equivalent	23.46 (22.56–24.40)	28.68 (25.32–32.29)	23.35 (22.43–24.29)	
More than high school	61.16 (59.62–62.66)	43.22 (39.09–47.45)	61.55 (60.02–63.06)	
Marital status (%)				< 0.001
Married/living with partner	64.63 (63.49–65.76)	57.94 (54.34–61.45)	64.78 (63.62–65.92)	
Never married	17.35 (16.32–18.42)	6.40 (4.56–8.91)	17.59 (16.55–18.68)	
Divorced, separated, or widowed	18.02 (17.38–18.68)	35.66 (32.54–38.91)	17.63 (16.99–18.30)	
PIR	3.03 (2.97–3.09)	2.31 (2.30–2.33)	3.05 (2.99–3.11)	< 0.001
PIR group (%)				< 0.001
< 1.3	24.45 (23.24–25.70)	37.71 (33.98–41.58)	24.16 (22.94–25.41)	
1.3–3.5	32.15 (31.08–33.25)	40.63 (36.33–45.08)	31.97 (30.88–33.07)	
> 3.5	43.40 (41.69–45.12)	21.66 (17.82–26.07)	43.88 (42.16–45.60)	
Current drinking (%)	2.53 (2.25–2.76)	2.45 (1.45–4.13)	2.53 (2.25–2.76)	0.899
Current smoking (%)	17.16 (16.33–18.02)	18.34 (15.61–21.44)	17.13 (16.28–18.01)	0.428
Height (cm)	168.89 (168.71–169.07)	167.49 (167.38–167.59)	168.92 (168.74–169.10)	0.513
Weight (kg)	82.56 (82.16–82.97)	89.52 (89.30–89.75)	82.41 (82.01–82.81)	< 0.001
BMI (kg/m^2)	28.86 (28.71–29.00)	31.81 (31.74–31.88)	28.79 (28.65–28.94)	< 0.001
WC (cm)	98.96 (98.59–99.34)	109.64 (109.46–109.81)	98.73 (98.36–99.09)	< 0.001
SBP (mmHg)	122.03 (121.70–122.35)	129.32 (129.13–129.52)	121.87 (121.55–122.19)	< 0.001
DBP (mmHg)	71.07 (70.74–71.40)	67.54 (67.41–67.66)	71.15 (70.82–71.48)	< 0.001
FPG (mmol/L)	5.47 (5.45–5.50)	6.64 (6.61–6.66)	5.45 (5.42–5.47)	< 0.001
PLT (10^9/L)	250.43 (248.97–251.89)	224.94 (224.23–225.65)	250.99 (249.51–252.47)	< 0.001
TC (mmol/L)	5.05 (5.03–5.07)	4.64 (4.63–4.65)	5.06 (5.03–5.08)	< 0.001
Triglycerides (mmol/L)	1.70 (1.67–1.72)	1.92 (1.90–1.93)	1.69 (1.67–1.72)	< 0.001
LDL‐C (mmol/L)	3.32 (3.30–3.34)	3.00 (2.99–3.01)	3.33 (3.31–3.35)	< 0.001
HDL‐C (mmol/L)	1.39 (1.38–1.40)	1.26 (1.26–1.27)	1.39 (1.38–1.40)	< 0.001
ALT (U/L)	25.45 (25.19–25.71)	25.79 (24.81–26.76)	25.45 (25.19–25.70)	0.550
AST (U/L)	25.29 (25.09–25.50)	27.42 (26.29–28.55)	25.24 (25.04–25.45)	0.002
Globulin (g/L)	28.40 (28.25–28.54)	29.45 (29.10–29.79)	28.37 (28.22–28.52)	< 0.001
Scr (μmol/L)	78.37 (77.96–78.79)	104.62 (104.23–105.00)	77.79 (77.40–78.19)	< 0.001
eGFR (ml/min/1.73 m^2)	94.31 (93.73–94.89)	69.12 (68.87–69.38)	94.87 (94.29–95.45)	< 0.001
Anti‐hypertension therapy (%)	25.84 (24.98–26.72)	72.46 (68.67–75.96)	24.82 (23.98–25.67)	< 0.001
Anti‐diabetic therapy (%)	6.83 (6.48–7.21)	25.35 (22.12–29.13)	6.42 (6.09–6.78)	< 0.001
Diagnosed diabetes (%)	8.75 (8.34–9.17)	37.70 (33.00–41.57)	8.11 (7.73–8.49)	< 0.001
Hypertension (%)	33.09 (32.17–34.03)	78.36 (74.76–81.58)	32.09 (31.19–32.98)	< 0.001
Diabetes (%)	13.22 (12.72–13.75)	45.39 (41.94–49.46)	12.51 (12.03–12.98)	< 0.001
SAFE	−23.62 (−25.94 to −21.29)	99.69 (98.58–100.80)	−26.34 (−28.65 to −24.03)	< 0.001

*Note:* Data were displayed as mean (95% confidence intervals) or numbers (95% confidence intervals) according to their data type.

Abbreviations: ALT, Alanine Aminotransferase; AST, Aspartate Aminotransferase; BMI, body mass index; DBP, diastolic blood pressure; eGFR, estimated glomerular filtration rate; FPG, fasting plasma glucose; HDL‐C, high density lipoprotein cholesterol; HF, heart failure; LDL‐C, low density lipoprotein cholesterol; PIR, poverty‐to‐income ratio; PLT, platelet; SAFE, steatosis‐associated fibrosis estimator; SBP, systolic blood pressure; Scr, serum cholesterol; TC, total cholesterol; WC, waist circumference.

* Comparison of categorical variables was performed by Chi‐square test. t‐test was used for the comparison of continuous variables.

**Figure 2:**
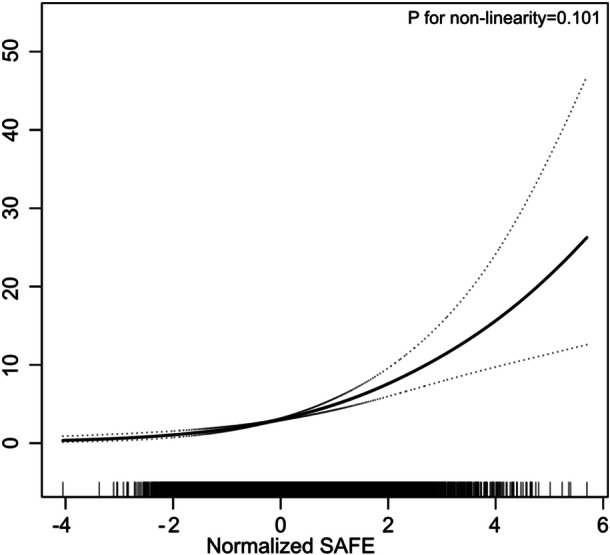
Smooth curve fitting illustrating the linear relationship between SAFE and prevalent HF. The model was adjusted for age, sex, race, education, marriage, current smoking and drinking status, BMI, WC, SBP, FPG, TC, triglycerides, HDL‐c, eGFR, anti‐hypertensive therapy, anti‐diabetic therapy, consistent with Model 2 in Table 2. The solid line represents the estimated risk of prevalent HF, while the dotted lines indicate the pointwise 95% confidence intervals. The association remained linear across the entire range of SAFE, with a *P* for non‐linearity > 0.05.

The logistic regression analysis results, presented in Table 2, demonstrated a significant association between SAFE and the risk of HF. In the crude model, each standard deviation (SD) increase in SAFE was associated with a 2.849‐fold increase in HF risk. After adjusting for demographic and lifestyle factors, including age, sex, race, education level, marital status, smoking, drinking, and PIR (Model 1), the risk was reduced to a 92.7% increase per SD. Further adjustments in Model 2, incorporating clinical variables such as BMI, WC, mSBP, FPG, TC, triglycerides, HDL‐c, eGFR, anti‐hypertensive therapy, and anti‐diabetic therapy, reduced the risk to 58.0% per SD increase. When SAFE was categorized into quartiles, individuals in Quartile 4 (highest SAFE scores) showed a 2.777‐fold increased risk of HF compared to those in Quartile 1 (lowest SAFE scores) under Model 2. A consistent upward trend was observed across quartiles, with *P* for trend < 0.001. These findings highlight the strong and independent relationship between higher SAFE scores and increased HF risk. To confirm this observed trend, a smooth curve fitting analysis was conducted (Figure 2), which showed a linear and steady increase in HF risk across the entire range of SAFE values, further validating the association. The results suggest that SAFE is a reliable indicator of prevalent HF risk, applicable across both continuous and categorical analyses, providing robust evidence of its utility in identifying individuals at higher risk for prevalent HF.

**Table 2:** Association between SAFE and the prevalent HF.

Variables	Odds Ratio (95% CI)					
	Crude	*p* value	Model 1	*p* value	Model 2	*p* value
SAFE (Per SD increase)	2.849 (2.625–3.093)	< 0.001	1.927 (1.748–2.124)	< 0.001	1.580 (1.277–1.955)	< 0.001
Quartiles of SAFE						
Quartile 1	Reference		Reference		Reference	
Quartile 2	3.540 (2.152–5.825)	< 0.001	2.110 (1.262–3.529)	0.005	1.689 (0.999–2.855)	0.051
Quartile 3	9.208 (5.644–15.021)	< 0.001	3.048 (1.782–5.214)	< 0.001	1.800 (1.035–3.131)	0.038
Quartile 4	38.760 (23.627–63.584)	< 0.001	7.559 (4.230–13.509)	< 0.001	2.777 (1.514–5.096)	0.001
*P* for trend		< 0.001		< 0.001		< 0.001

*Note:* Crude: no adjustment; Model 1: adjusted for age, sex, race, education, marriage, current smoking and drinking status, PIR; Model 2: Model 1 + BMI, WC, SBP, FPG, TC, triglycerides, HDL‐c, eGFR, anti‐hypertensive therapy, anti‐diabetic therapy.

**Figure 3:**
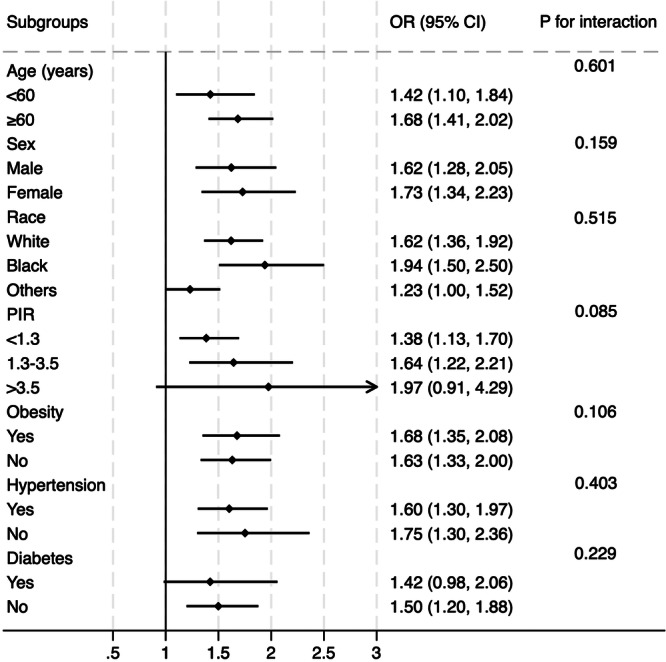
Subgroup analysis of the correlation between SAFE and prevalent HF. The multivariate logistic model was adjusted for all variables included in Model 2 of Table 2, except the variable used to define each subgroup. The association remained consistent across subgroups defined by sex, age, race, diabetes, hypertension, and obesity.

To further validate the findings observed in the overall population, subgroup analyses were conducted across various conventional subpopulations (Figure 3). The logistic regression models were adjusted for all covariates included in Model 2, except for those used to define the subgroups. The results demonstrated that the relationship between SAFE and the prevalence of HF was consistent across different subgroups, including those categorized by sex, age, race, PIR, diabetes, hypertension, and obesity. No significant interactions were found between SAFE and these subgroup variables (all *P* for interaction > 0.05), confirming the robustness of the association.

The detecting value of SAFE for HF was analyzed using ROC and reclassification methods (Table 3). The ROC analysis showed that the AUC for SAFE alone was 0.804 (95% CI: 0.799–0.808). Incorporating SAFE into the clinical risk factors (variables from Model 2 in Table 2) resulted in an increase in AUC from 0.873 to 0.876, with this improvement reaching statistical significance (*p* < 0.001). Additionally, reclassification analysis highlighted the added detecting value of SAFE, with a continuous NRI of 0.137 (95% CI: 0.074–0.199, *p* < 0.001) and an IDI of 0.003 (95% CI: 0.002–0.005, *p* < 0.001). These findings suggest that SAFE could contribute meaningfully to enhancing the detection accuracy of clinical models, reinforcing its role as a valuable addition for HF risk stratification.

**Table 3:** Assessment of the value of SAFE for detecting prevalent HF.

Model	AUC (95% CI)	*p* value	P for Comparison	NRI (continuous)	*p* value	IDI	*p* value
SAFE	0.804 (0.799–0.808)	< 0.001	—	—	—	—	—
Clinical risk factors^a^	0.873 (0.869–0.876)	< 0.001	—	—	—	—	—
Clinical risk factors + SAFE	0.876 (0.872–0.879)	< 0.001	< 0.001	0.137 (0.074–0.199)	< 0.001	0.003 (0.002–0.005)	0.001

^a^ Clinical risk factors: age, sex, race, education, marriage, current smoking and drinking status, PIR, BMI, WC, SBP, FPG, TC, triglycerides, HDL‐c, eGFR, anti‐hypertensive therapy, anti‐diabetic therapy.

## 4 Discussion

In this investigation, a pronounced positive link emerged between SAFE—an indicator of liver fibrosis correlated to NAFLD—and the likelihood of existing HF in a general population. Across the entire spectrum of SAFE measurements, the relationship appeared linear, suggesting that higher SAFE values were associated with lower HF risk. This finding persisted even after adjusting for standard cardiovascular risk elements, demonstrating its stability in various subgroups. Moreover, when SAFE was integrated alongside conventional cardiovascular risk factors, both ROC and reclassification indices showed notable improvements, underscoring SAFE's potential to strengthen the detection of prevalent HF.

### 4.1 Linear and Robust Correlation Between Safe and the Risk of Prevalent HF

This analysis provides strong evidence supporting the link between SAFE and the presence of HF, highlighting SAFE's potential utility in detecting HF in the general population. Initially, multivariate logistic regression was used to explore this association, controlling for various demographic, clinical, anthropometric, and medical history factors. The results demonstrated an independent connection between SAFE and prevalent HF, which was not influenced by traditional cardiovascular risk factors. To assess the nature of the relationship, a smooth curve fitting technique was employed, revealing that the connection between SAFE and prevalent HF appeared nearly linear across the full range of SAFE values. This linearity suggests that higher SAFE values were consistently associated with a lower likelihood of prevalent HF, without indication of a plateau or threshold. Lastly, a subgroup analysis was conducted to verify the consistency of the findings across different cardiovascular risk groups, including those defined by age, gender, ethnicity, socioeconomic status, diabetes, hypertension, and obesity. The analysis affirmed that the results were robust across these subgroups, further supporting the broader applicability of the relationship between SAFE and prevalent HF.

### 4.2 Value of SAFE in Identifying Prevalent HF

In the subsequent segment of our statistical evaluation, we investigated whether SAFE could enhance the identification of prevalent HF within the general population. To approach this, we utilized both ROC and reclassification analyses, providing diverse perspectives on SAFE's diagnostic capabilities. The assessment revealed that SAFE alone yielded a modest AUC for detecting HF. However, when SAFE was integrated with established cardiovascular risk factors, the predictive performance of the model saw a significant improvement, indicating that SAFE may play a crucial role in refining HF detection strategies. Despite these promising results, it is essential to acknowledge that ROC analysis evaluates the combined efficacy of SAFE and traditional risk factors without isolating SAFE's individual impact [18, 19]. This phenomenon means that the specific contribution of SAFE to the overall model performance might be either overestimated or underestimated, thereby offering limited clarity on the actual value of incorporating SAFE alongside conventional cardiovascular indicators in detecting prevalent HF.

To address the shortcomings of ROC analysis in evaluating new biomarkers, researchers have turned to reclassification methods that utilize metrics like NRI and IDI [20, 21, 22]. ROC analysis measures a model's overall ability to discriminate between cases (e.g., individuals with a disease) and controls (those without) by plotting the true positive rate (sensitivity) against the false positive rate (1‐specificity) across various threshold settings. The primary metric derived from ROC analysis is the AUC, which provides a single summary value indicating the model's discriminative power; an AUC of 1 signifies perfect discrimination, while an AUC of 0.5 suggests no better than random chance. However, ROC analysis offers a broad assessment and may not capture specific improvements in risk prediction relevant to clinical decision‐making. In contrast, reclassification analysis focuses on the incremental value that a new biomarker adds to an existing model by examining how individuals are reclassified into different risk categories when the biomarker is included [23]. This approach uses metrics such as NRI and IDI to quantify how well the new biomarker improves the accuracy of risk stratification, thereby providing more detailed insights into the practical benefits of adding the biomarker. Reclassification analysis is particularly useful for understanding changes in risk predictions that can directly impact clinical decisions and patient management. However, it has limitations, including dependence on predefined risk categories and variability in baseline models across studies, which can make comparisons challenging [24]. In summary, while ROC analysis provides a comprehensive measure of a model's overall discriminative ability, reclassification analysis offers a more nuanced evaluation of how a new biomarker enhances risk prediction and classification, making the two methods complementary in assessing model performance.

In our research, the integration of SAFE into the existing cardiovascular risk factor model led to notable enhancements in both continuous NRI and IDI. These improvements signify a more precise identification of individuals with prevalent HF. The favorable outcomes were corroborated through both ROC and reclassification analyses, underscoring the robustness of the findings. Consequently, the addition of SAFE to the cardiovascular risk assessment framework appears to significantly bolster the detection capabilities for prevalent HF within the general population. This evidence strongly supports the potential of SAFE as a valuable component in refining HF identification strategies, ultimately contributing to better clinical outcomes and more targeted interventions.

### 4.3 Mechanisms Underlying the Association Between SAFE and HF

**Figure 4:**
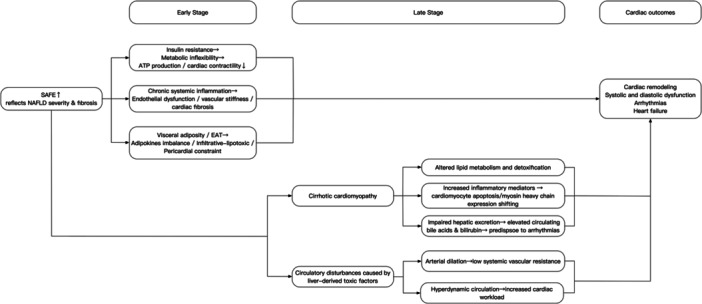
Potential pathophysiological pathways linking SAFE Score to HF.

The underlying mechanisms linking the SAFE score with HF appear to be multifactorial, spanning early metabolic dysfunction to advanced cirrhosis‐related cardiac complications (Figure 4). At the population level, an elevated SAFE score often reflects the severity of NAFLD and hepatic fibrosis. NAFLD is strongly associated with insulin resistance, visceral adiposity, and systemic low‐grade inflammation, all of which are well‐established contributors to cardiovascular disease [25]. Insulin resistance disrupts myocardial energy metabolism by reducing glucose uptake and forcing excessive reliance on fatty acid oxidation [26]. This shift increases oxygen demand and promotes mitochondrial dysfunction, while toxic lipid intermediates induce oxidative stress and apoptosis. As a result, ATP generation becomes inefficient, impairing myocardial contractility and diastolic relaxation. These metabolic disturbances foster cardiac remodeling and predispose to HF. Systemic low‐grade inflammation represents another critical pathway [6]. NAFLD is characterized by elevated circulating cytokines such as tumor necrosis factor‐α (TNF‐α) and interleukin‐6, which promote endothelial dysfunction, vascular stiffness, and a pro‐fibrotic myocardial environment [27]. Chronic activation of inflammatory signaling exacerbates cardiomyocyte apoptosis and interstitial fibrosis, ultimately impairing ventricular compliance and facilitating the transition to clinical HF. Visceral adiposity, which is common in NAFLD, increases the risk of HF in several ways. Visceral fat tissue releases adipokines, such as leptin, while lowering protective adiponectin [28]. These changes make insulin resistance worse and promote chronic inflammation. Furthermore, the infiltrative‐lipotoxic hypothesis proposes that epicardial adipose tissue (EAT), frequently increased in NAFLD and obesity, progressively infiltrates the adjacent myocardium. This process disrupts cardiac ultrastructure and alters electrophysiological properties, resulting in left ventricular hypertrophy, diastolic dysfunction, and arrhythmogenic substrate [29]. Additionally, the pericardial constraint hypothesis proposes that excess EAT impairs cardiac filling by exerting mechanical compression on the myocardium, resembling constrictive pericarditis [30].

In advanced stages of NAFLD‐related fibrosis, cirrhotic cardiomyopathy may provide an additional link to HF. Cirrhotic cardiomyopathy affects up to half of patients with cirrhosis and is characterized by reduced pumping reserve, impaired diastolic relaxation, and abnormalities in cardiac electrical activity [31, 32]. The pathophysiology is multifactorial. Cirrhosis alters lipid metabolism and the detoxification of drugs, ethanol, and hormones [13]. In addition, inflammatory mediators—including nitric oxide, carbon monoxide, endocannabinoids, and cytokines such as TNF‐α—trigger negative inotropic responses and promote cardiomyocyte apoptosis. These processes drive a shift in myosin heavy chain expression from the stronger α isoform to the weaker β isoform, ultimately reducing myocardial contractility [33]. Impaired hepatic excretion further results in elevated circulating bile acids and bilirubin, which interfere with sinus rhythm, depress myocardial activity, and predispose to arrhythmias [34]. Hemodynamic disturbances also play a critical role: liver‐derived toxic factors cause arterial dilation and hyperdynamic circulation, lowering systemic vascular resistance and increasing cardiac workload [33]. As liver failure advances and arterial dilation worsens, the heart's systolic reserve is eventually depleted; At that point, the heart cannot further increase cardiac output, resulting in arterial underfilling and a decreased effective circulating volume [35].

The above mechanisms provide plausible biological explanations for the observed association between SAFE and HF risk in community‐based populations. Current evidence suggests that insulin resistance, inflammation, visceral adiposity, and, in advanced stages, cirrhotic cardiomyopathy and circulatory disturbances may explain the association between SAFE and HF. These mechanisms link NAFLD and hepatic fibrosis to cardiac dysfunction, but the precise biological pathways remain incompletely understood. Further research is needed to clarify the underlying processes and confirm how SAFE captures metabolic and inflammatory changes related to HF risk.

### 4.4 Clinical Implications

The primary clinical implication of this study is the enhanced understanding of the relationship between SAFE and prevalent HF, reinforcing the established link between NAFLD and the development of HF. Given that NAFLD affects a significant portion of the population and is a known risk factor for cardiovascular diseases, SAFE—a quantified score of NAFLD‐associated hepatic fibrosis—emerges as a valuable tool for improving HF identification within the general population. Its cost‐effectiveness makes SAFE particularly advantageous for integration into clinical practice, especially in primary care settings where early detection is crucial. This potential benefit may be most pronounced in rural or resource‐limited areas, where access to advanced diagnostic tools for HF (e.g., echocardiography or natriuretic peptide testing) may be limited. In such contexts, incorporating SAFE into routine assessments could provide an accessible approach to triage patients who may warrant prompt confirmatory evaluation or referral. By incorporating SAFE into routine screenings, healthcare providers can more accurately identify patients at risk for HF at earlier stages, facilitating timely interventions and management strategies. This standardized scoring system enables general practitioners to deliver personalized care tailored to individual patients’ dietary and lifestyle behaviors, addressing the critical need for improved HF management in primary care. Additionally, the adoption of SAFE can help bridge existing gaps in HF detection, potentially reducing hospitalizations and improving patient outcomes. However, successful implementation may require training and integration with electronic health records to ensure seamless use. Future research should focus on validating SAFE across diverse populations and exploring its long‐term predictive capabilities. Ultimately, incorporating SAFE into cardiovascular risk assessments can lead to more proactive and preventive healthcare, enhancing the quality of life for patients and alleviating the burden of heart failure on healthcare systems.

### 4.5 Limitations

This study presents several limitations that should be acknowledged when interpreting the results. Firstly, the cross‐sectional design inherent to the NHANES restricted our ability to infer causality between SAFE and prevalent HF. As data were collected at a single time point, it is impossible to determine the temporal sequence of SAFE scores and the onset of HF, limiting our understanding of whether elevated SAFE leads to HF or vice versa. Secondly, a large number of NHANES subjects were excluded due to missing data. As illustrated in Figure 1, a significant part of the exclusion was due to an age less than 20 years (*n* = 35,522), these subjects were teenagers, which may show significant differences in lifestyle, laboratory, anthropometric, and medical history data compared to adult participants. More importantly, many parameters could change when these teenagers become adults. Therefore, our current analysis firstly excluded these subjects. However, there were still 11,224 adult subjects who were further removed due to a lack of related data. To assess the potential impact of this exclusion, we added Table S1 to compare basic characteristics (age, sex, and race) between the final analytic sample and all the subjects aged ≥ 20 years old. The demographic profile was highly comparable between the two groups, suggesting that selection bias due to missing data could be minimal. Thirdly, despite NHANES's comprehensive sampling methods, there remains a potential for selection bias, as specific demographic groups may be underrepresented, or non‐respondents might differ systematically from respondents. This limitation could affect the generalizability of our findings to the broader population. Additionally, the study relies partly on self‐reported data for variables such as medical history and lifestyle factors, which are susceptible to recall bias and social desirability bias, potentially compromising the accuracy of the information. Another limitation is the possibility of residual confounding; although we controlled for numerous demographic, laboratory, anthropometric, and medical history variables, unmeasured or inadequately measured confounders may still influence the observed association between SAFE and HF. Lastly, while SAFE demonstrated its potential as a tool for HF identification, its effectiveness across different populations and clinical settings requires further validation through longitudinal studies and diverse cohorts. These limitations underscore the need for cautious interpretation of the results and highlight areas for future research to elucidate the relationship between SAFE and HF better.

## 5 Conclusion

The current study identified a nearly linear and robust relationship between SAFE and prevalent HF, suggesting that SAFE may serve as a useful indicator for enhancing HF detection in the general population.

## Author Contributions

Wenrui Shi and Yuancong Wu designed the study, Jieun Park and Wenrui Shi analyzed the data, Wenrui Shi and Yangbin Shi wrote the manuscript. Yuancong Wu read and proofed the manuscript.

## Funding

The authors have nothing to report.

## Ethics Statement

Every subject in the NHANES study provided a written consent for participating the survey. The NCHS Institutional Ethics Review Board approved the study design of NHANES. Therefore, additional Ethics Review is unnecessary for the current analysis.

## Consent

Before participating in the study, all participants signed up with informed consent.

## Conflicts of Interest

The authors declare no conflicts of interest.

## Supporting information


**Table S1:** Basic characteristic profile before and after exclusion for missing covariates.

## Data Availability

The data used in the current study could be acquired from Yuancong Wu with a reasonable request. The data could also be downloaded from the NHANES official website. (https://www.cdc.gov/nchs/nhanes/index.html).
