# Supplementary material for: Enhancing Heart Failure Detection in the General Population Using the Steatosis‐Associated Fibrosis Estimator
Source: Clin Cardiol. 2026 Apr 20;49(4):e70318. doi: 10.1002/clc.70318 (PMC13094360; doi:10.1002/clc.70318)
Supplement: Supplementary file 1 — Table S1: Basic characteristic profile before and after exclusion for missing covariates. [file CLC-49-e70318-s001.docx]

Table S1. Basic characteristic profile before and after exclusion for missing covariates.

| Variables | Subjects aged ≥20 years (44,790) | Finally enrolled subjects (33,566) |
| --- | --- | --- |
| Age (years) | 47.25 (46.85-47.65) | 46.90 (46.47-47.32) |
| Male (%) | 48.08 (47.62-48.55) | 48.73 (48.02–49.26) |
| Race (%) |  |  |
| Mexican American | 8.42 (7.26-9.75) | 8.22 (7.05–9.56) |
| Other Hispanic | 5.35 (4.64-6.18) | 5.04 (4.32–5.86) |
| Non-Hispanic white | 67.28 (64.86-69.62) | 69.29 (66.92–71.57) |
| Non-Hispanic black | 11.39 (10.18-12.73) | 10.32 (9.18–11.59) |
| Others | 7.55 (6.86-8.31) | 7.13 (6.49–7.83) |
